# Home Health Care Practices to Reduce Acute-Care Hospitalizations

**DOI:** 10.1093/geroni/igaa057.449

**Published:** 2020-12-16

**Authors:** Bonnie Albright, Yael Pelenur

**Affiliations:** 1 University of Massachusetts - Boston, Boston, Massachusetts, United States; 2 University of Vermont, Burlington, Vermont, United States

## Abstract

Avoidable acute-care admissions are linked to negative health outcomes for patients and are costly to insurers. Home health care agencies (HHAs) are important players in preventing these admissions. Research on best practices to prevent acute-care admissions in the home health care context is limited. The purposes of this study were both to discover what practices HHAs are implementing in Medicare-certified home health care episodes to prevent acute-care admissions, and to learn what barriers HHAs face in implementing these practices. This study used mixed methods including qualitative and quantitative elements. Seven key informant interviews were used to develop a web survey that was emailed to all Medicare-certified HHAs in Massachusetts (response rate 12.43%, n=23). Using qualitative methods including thematic review, open-coding, and member-checking this study developed a four-categorization method. The categories are assessment, interventions, communication, and global practices. The study also developed a taxonomy for describing barriers to implementing practices. The distribution of responses for the new taxonomic categories were: patient-related (32.35%), staffing-related (29.41%), software-related (17.65%), physician/hospital-related (14.29%), and reimbursement/regulation-related (5.88%). This study fills a gap in research by describing the realities of home health care practice in the context of avoidable acute-care admissions. The categorization of best practices and the taxonomy of barriers developed in this study provide frameworks for understanding HHA practice. Further research is needed to effectively reduce avoidable acute-care admissions during and after Medicare-certified home health care episodes.

